# Classification of ALS molecular subtypes: a literature review on machine learning applications and their clinical value

**DOI:** 10.1186/s12916-026-04725-y

**Published:** 2026-02-23

**Authors:** John K. Jammal, Esteban A. Gomez, Ammar Al-Chalabi, Alfredo Iacoangeli

**Affiliations:** 1 Department of Biostatistics & Health Informatics, Institute of Psychiatry Psychology & Neuroscience, King’s College London, 16 De Crespigny Park, London, SE5 8AB UK; 2 Department of Basic and Clinical Neuroscience, Institute of Psychiatry Psychology & Neuroscience, King’s College London, 5 Cutcombe Rd, London, SE5 9RX UK; 3 Ground RR Block QE II Medical Centre Ralph & Patricia Sarich Neuroscience Building, Perron Institute for Neurological and Translational Science, 8 Verdun St, Nedlands, WA 6009 Australia; 4 NIHR Maudsley Biomedical Research Centre (BRC), South London and Maudsley NHS Foundation Trust, 16 De Crespigny Park, London, SE5 8AF UK

**Keywords:** Amyotrophic lateral sclerosis, Molecular subtypes, Machine learning, Oxidative stress, Glial cell activation, Transposable elements, Transcription dysregulation, Precision medicine

## Abstract

**Background:**

Amyotrophic lateral sclerosis (ALS) is a fatal neurodegenerative disease characterised by considerable heterogeneity in both its underlying biological mechanisms and clinical presentation. High-dimensional transcriptomic datasets offer an opportunity to characterise this variation at the molecular level; however, traditional statistical methods struggle with their scale and complexity.

**Main body:**

Machine learning approaches can reduce dimensionality and uncover latent patterns, enabling the identification of molecular subtypes that may refine prognosis and support patient stratification. Recent transcriptomic studies employing unsupervised machine learning have identified ALS subtypes with distinct molecular and clinical characteristics. Redefining ALS into more homogeneous molecular and clinical subtypes could transform all areas of ALS research by supporting novel experimental designs and precision medicine approaches.

**Conclusions:**

In this review, we summarise and critically assess these studies, discussing their findings, strengths, and limitations, and highlighting research gaps and challenges that must be addressed to enable their translation into biomedical and clinical practice.

**Supplementary Information:**

The online version contains supplementary material available at 10.1186/s12916-026-04725-y.

## Background

The biological and clinical heterogeneity of amyotrophic lateral sclerosis (ALS) [1] greatly impacts our efforts in understanding its pathogenesis and developing treatments by acting as a major confounding factor in studies and limiting the effectiveness of clinical procedures. Previous research into the genetics of people with ALS (pwALS) has uncovered a number of genetic contributors to disease pathogenesis including rare mutations and common polymorphisms in many genes, among which *SOD1*, *C9orf72*, *FUS*, and *TARDBP* can causally explain the largest proportion of patients [2–7]. Despite these advances, large effect variants only causally explain about 20% of ALS [8, 9]. As a result, a re-classification of ALS based on our current knowledge of disease-associated gene variants would not be widely applicable. This has prompted researchers to look beyond single-gene approaches and toward more comprehensive molecular profiling strategies that can capture the complex and variable processes underlying all ALS and dissect the disease heterogeneity. Technological advances in high throughput molecular profiling have led to the identification of various molecular subtypes of ALS utilising unsupervised machine learning (ML) models to analyse the ALS transcriptome. Most studies implementing such an approach identify a range of molecular ALS subgroups consistent with alterations in distinct biological processes such as transposable element (TE) expression (ALS-TE), oxidative stress (ALS-Ox), neuroinflammation, and increase in astrocyte activity (ALS-Glia) [10–13].

This literature review will explore the findings across recent studies on the identification of molecular subtypes of ALS and their potential clinical relevance while touching upon their methodological differences. An in-depth overview of the various methodologies and experimental designs is available in Additional file 1.

## Main text

### Chronology of identification of ALS molecular subtypes in the ALS Transcriptome

**Fig. 1:**
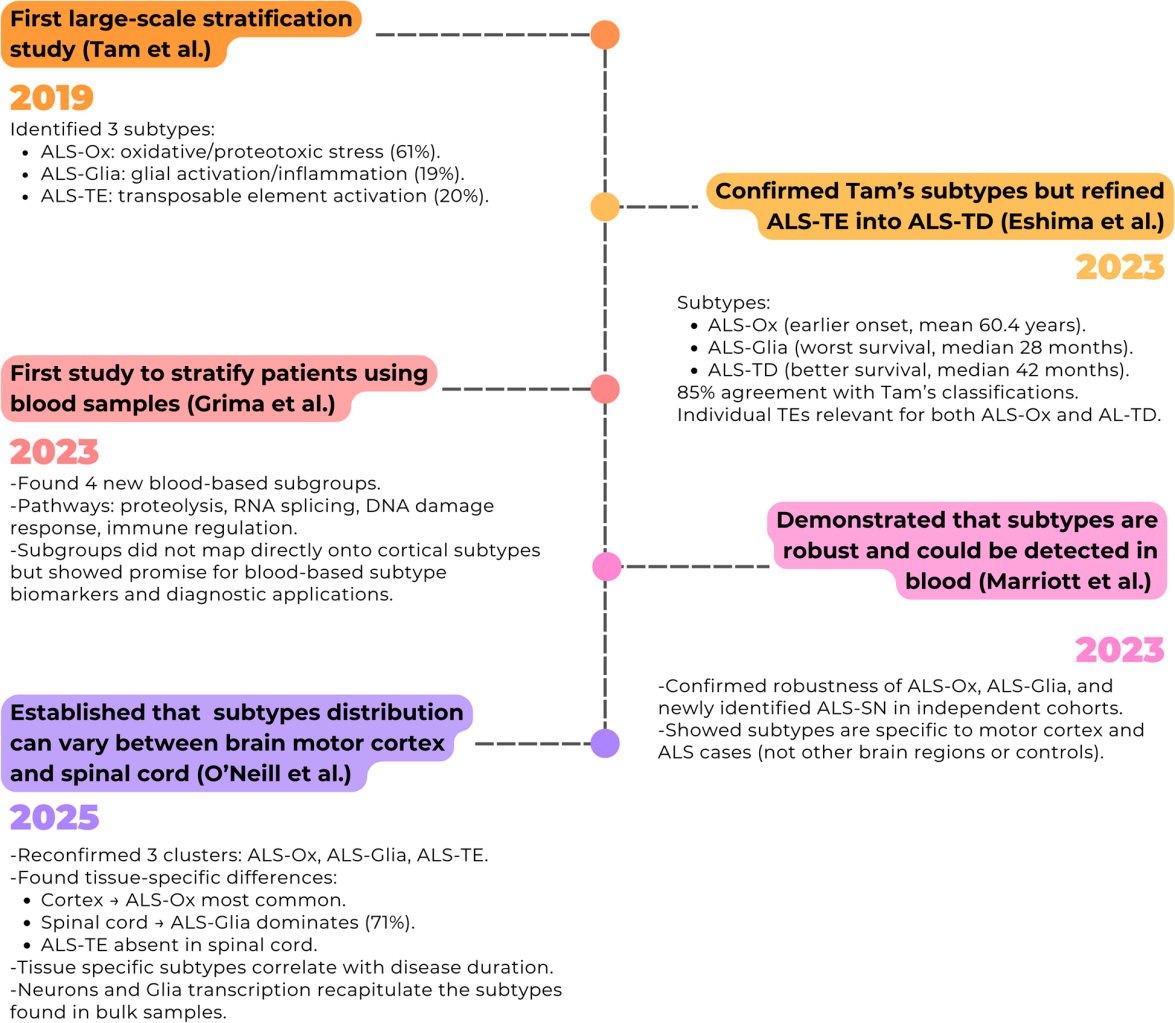
Timeline summarising key studies that investigated molecular subtypes of ALS using stratification approaches. Early work by Tam et al. [13] identified three cortical subtypes (ALS-Ox, ALS-Glia, ALS-TE), which were later refined and validated by Eshima et al. [10]. Subsequent studies in 2023 extended these findings to blood-based stratification [11, 14], highlighting potential biomarker applications, while also demonstrating subtype robustness in independent cohorts. Most recently, O’Neill et al. [12] revealed tissue-specific distributions between cortex and spinal cord, suggesting spatial heterogeneity in subtype prevalence

Research exploring the molecular subtypes of ALS using ML and transcriptomics is relatively recent (Fig. 1), with the first large-scale molecular stratification study published in 2019 [13]. Using unsupervised ML to analyse the NYGC ALS Consortium transcriptomic data of 148 post-mortem cortex samples of 77 pwALS, they identified three distinct subtypes. The largest (61% of patients) was characterised by alterations in oxidative and proteotoxic stress processes, while a second (19%) showed predominant glial activation and inflammatory signatures. A third group (20%) displayed elevated transposable element expression and depletion of pathways normally regulated by TDP-43, thereby linking this subtype to TDP-43 dysfunction [13]. Following this initial study, several studies have explored transcriptome-based stratification in ALS, not only seeking to replicate these findings but also exploring the various potential applications this method can provide, such as integrating additional datasets and tissue types, applying alternative analytical methods, or assessing the prognostic and clinical significance of these subtypes.

Utilising 451 samples from 208 pwALS, another study [10] increased the sample size, investigating the relationship between molecular subtypes and clinical outcomes using patient survival and age of onset. They were able to find three molecular subtypes consistent with the ones found in the 2019 study, despite some differences partially due to different methodologies for the characterisation of subgroups. Indeed, considering the 140 samples shared between the two studies, they observed 85% agreement in sample classification. However, the larger sample size and methodological differences contributed to the redefinition of the ALS-TE subgroup into the ALS-transcriptional dysregulation subtype (ALS-TD), and the upregulation of specific TEs was found in samples belonging to both ALS-TD and ALS-Ox groups. Notably, the ALS-TD subtype reflected many of the hallmark pathways of TDP-43 dysfunction, including impaired transcriptional regulation, cryptic exon–associated nonsense-mediated decay, and aberrant expression of transposable and non-coding RNAs, suggesting that TDP-43 pathology underlies its transcriptomic profile [10]. Clinically, individuals with ALS-TD had a better prognosis, with a median survival of 42 months (Table 1). By contrast, ALS-Ox was associated with an earlier age of onset and intermediate survival (mean age of onset 60.4 ± 1.16 years and median survival 36 months), while ALS-Glia displayed the worst prognosis, with a median survival of 28 months and an average age of onset of 63.2 ± 1.83 years (Table 1).

**Table 1:** Molecular subtypes of ALS identified across studies

Study	Tissue type	Subtype	Cell types	Proportion of pwALS	Replicated in independent samples	Tested on pre-mortem samples	Molecular characteristics	Clinical feature correlation	Code and data availability
Tam et al., 2019 [13]	**Frontal and motor cortex tissue**	**Increased transposable element expression** **(ALS-TE)**	**n/a**	20%	Yes	No	↑ Retrotransposon activation ↑ TDP-43 dysfunction ↑ Transposable elements ↓ Spliceosome components ↓ Protein export pathways	Associated with limb onset (**56% of patients**) No survival differences or significant correlations	**Data:** All data is provided by the Gene Expression Omnibus database: Motor cortex RNA-seq datasets from CSHL motor cortex: **Accession Numbers:** **GSE122649** CLIP-seq and RNA-seq datasets from SH-SY5Y cells: **Accession Numbers:** **GSE122650** RNA-seq datasets provided by the NYGC ALS Consortium: **Accession Numbers:** **GSE124439** ***Code is unavailable***
		**Oxidative stress** **(ALS-Ox)**	**n/a**	61%	Yes	No	↑ Oxidative stress markers ↑ Proteotoxic stress ↑ Autophagy pathways ↑ Oxidative phosphorylation	No survival differences or significant correlations	
		**Glial markers** **(ALS-Glia)**	- Astrocytes (markers) - Microglia (markers) Oligodendrocytes (markers)	19%	Yes	No	↑ Glial activation markers ↑Neuroinflammation	No survival differences or significant correlations	
Eshima et al., 2023 [10	**Frontal and motor cortex tissue**	**Dysregulation in Transcription** **(ALS-TD)**	n/a	29.1%	No	No	↑ Transcriptional dysregulation ↑ Pseudogenes ↑ lncRNAs, miRNAs ↑ Nonsense-mediated decay	**Better prognosis** Median survival: 42 months Mean age of onset: (62.7 ± 1.68 years)	**Data:** Raw data from NCBI Run Selector: Accession code: **PRJNA644618** The RSEM processed gene count matrix from Gene Expression Omnibus: Accession code: **GSE153960** Processed RNA-seq count files: https://figshare.com/authors/Jarrett_Eshima/13813720 **Code:** Code for the analysis available in the Barbara Smith Lab GitHub repository: https://github.com/BSmithLab/ALSPatientStratification Scripts used for supervised Classification: https://github.com/plaisier-lab/U5_hNSC_Neural_G0 **Classification models are unavailable**
		**Oxidative stress** **(ALS-Ox)**	**Cell Deconvolution analysis:** - Excitatory neurons - Inhibitory neurons	53.2%	No	No	↑ Oxidative stress ↓ Oxidative phosphorylation ↑ Proteotoxic stress ↑ Synaptic alterations	Intermediate survival: 36 months Mean age of onset: (60.4 ± 1.16 years)	
		**Glial markers** **(ALS-Glia)**	**Cell Deconvolution analysis:** - Microglial - Glial progenitor - Vascular cell - Inhibitory neurons	17.7%	No	No	↑ Glial activation ↑ Neuroinflammation ↑ MHC Class II ↑ Complement cascade ↓ Transposable elements	**Worse prognosis** Median survival: **28 months** Mean age of onset: (63.2 ± 1.83 years)	
Grima, et al., 2023 [14]	**Peripheral blood tissue**	**Cluster 0**	n/a	44%	No	n/a	↑ Translation and adaptive immune response ↓ Inflammatory/innate immune response	n/a	**Data:** Raw Fastq files, raw gene counts and offset matrix are available at NCBI Gene Expression Omnibus: Accession code: **GSE234297** **Code:** Code for the analysis is available in the Gitlab repository: https://gitlab.com/mq-mnd/grp_williams/sals_blood_rnaseq
		**Cluster 1**	n/a	~ 10.5%	No	n/a	↓ Proteolysis, metabolic and RNA-splicing pathways	n/a	
		**Cluster 2**	n/a	~ 10.5%	No	n/a	↑ Proteolysis, metabolic and RNA-splicing pathways (opposite to cluster 1)	n/a	
		**Cluster 3**	n/a	35%	No	n/a	Intermediate profile between clusters 1 and 2 with few unique genes	n/a	
Marriott, et al., 2023 [11]	**Frontal and motor cortex tissue**	**Synaptic and neuropeptide signalling** **(ALS-SN)**	**Cell deconvolution analysis:** - Motor neurons	53.6%	Yes	No	↑ Synaptic signalling ↑ Neuropeptide activity ↑ Mitochondrial ATP synthesis ↑ cAMP signalling ↑ Neuroactive ligand binding	Youngest mean age of onset: (**58.8 ± 11.6**) Disease duration in years (median (IQR)): 3.16 (1.96)	**Data:** The RNA- seq validation set generated by Zucca et al. [15]can be found via the NCBI Sequence Read Archive: **Accession numbers:** **PRJNA416880** **PRJNA474387** The gene expression microarray data generated by Van Rheenan [16] are available via the Gene Expression Omnibus database: **Accession number:** **GSE112681** The London Neurodegenerative Diseases Brain Bank data is available upon reasonable request Target ALS dataset is available upon approval by the TargetALS Post-mortem Tissue Core **Code:** The code used for the analyses performed: https://github.com/KHP-Informatics/HierarchicalClusteringALS/ **Class Assignment models are available to use via the link**: https://alsgeclustering.er.kcl.ac.uk
		**Oxidative stress and apoptosis** **(ALS-Ox)**	**Cell deconvolution analysis:** - Astrocytes - Endothelial cells	25%	Yes	No	↑ Oxidative stress ↑ Apoptosis signalling ↑ Muscle contraction ↑ Anti-inflammatory processes ↑ Metalloproteinase activity	Oldest mean age of onset: (65.7 ± 12.3) Disease duration in years (median (IQR)): 2.30 (1.81)	
		**Glial markers** **(ALS-Glia)**	**Cell deconvolution analysis:** - Microglial - Oligodendrocytes	21.4%	Yes	No	↑ Neuroinflammation ↑ MHC class II complex ↑ Complement cascade ↑ Interferon signalling ↑ M1 activated microglia	Mean age of onset: (61.7 ± 15.7) Disease duration in years (median (IQR)): 2.38 (1.75) No significant correlations	
O’Neill et al., 2025 [12]	**Frontal and motor cortex tissue**	**Increased transposable element expression** **(ALS-TE)**	**Single cell composition:** - L5ET neurons (TDP-43 dysfunction) - Excitatory neurons - Inhibitory neurons	20%	No	No	↑ TDP-43 pathology L5ET Neurons ↑Transposable elements expression in L5ET Neurons ↑ TDP-43 splicing defects	↓Disease duration against Eigengene score (***r* = − 0.25, *P* < 0.05**)	**Data:** The RNA- seq and snRNA- seq data are available via the Gene Expression Omnibus: **Accession Numbers:** **GSE271156** Post-mortem frontal/motor cortex and spinal cord RNA- seq was provided by the NYGC ALS Consortium: **Accession Numbers:** **GSE137810** **Code:** The software developed that can quantify transposable elements from single-cell and single-nuclei datasets: https://github.com/mhammell-laboratory/CellRangerTE **Class Assignment DANCer model is available via the study’s GitHub link**: https://github.com/mhammell-laboratory/DANcer
		**Oxidative stress** **(ALS-Ox)**	**Single cell Composition:** - L5ET neurons	70%	No	No	↑Oxidative stress ↑Mitochondrial dysfunction	No significant correlations	
		**Glial markers** **(ALS-Glia)**	**Single cell composition:** - L5ET neurons - Microglial	10%	No	No	↑Neuroinflammation ↑Microglial activation	No significant correlations	
	**Spinal Cord**	**Oxidative stress** **(ALS-Ox)**	**n/a**	29%	No	No	↑Oxidative stress ↑Neuroactive ligands	↓ Disease duration against Eigengene score (***r* = − 0.29, *P* < 3e − 5**)	
		**Glial markers** **(ALS-Glia)**	- Astrocytes (markers) - Microglia (markers)	71%	No	No	↑Inflammatory signatures ↑ TDP-43 dysfunction	↓Disease duration against Eigengene score (***r* = − 0.35, *P* < 2e − 10**)	

The arrows indicate increase or decrease in activity of the characteristic in question (↑ indicates increase in activity; ↓ indicates decrease in activity). Values in bold under the Clinical feature correlation column represent significant outcomes

Building on these findings, a further study in 2023 [14] investigated if molecular subtypes could also be identified and distinguished de novo from whole peripheral blood RNA-seq data. Using samples from 96 people with sporadic ALS (sALS), they applied unsupervised K-means clustering and identified four patient subgroups. The gene expression profiles of these clusters were also explored; differential gene expression analysis between clusters identified 5756 subgroup-defining genes with most differentially expressed in two clusters relative to all other clusters. Furthermore, the study also used GO biological pathway and KEGG pathway enrichment of these subgroup-defining genes and identified pathways involving proteolysis, RNA splicing, DNA damage response, and immune regulation, suggesting that peripheral signatures may reflect heterogeneous systemic responses to ALS. Unlike earlier studies that stratified post-mortem motor cortex tissue, this work focused on whole blood, a minimally invasive and clinically accessible biospecimen. Although the resulting subgroups cannot be directly aligned with the established cortical molecular subtypes identified in other studies, their work demonstrates the potential of blood-based transcriptomics for patient stratification and lays a foundation for developing accessible biomarkers that could be monitored longitudinally throughout disease progression.

Another study published in 2023 [11] further investigated the robustness and clinical applicability of the ALS molecular subtypes across independent cohorts. Using post-mortem motor cortex ALS samples from the London Neurodegenerative Diseases Brain Bank as a discovery cohort (*n* = 112) [17, 18], they validated the three cortical molecular subtypes in an independent dataset from TargetALS (*n* = 93 sALS samples). To test tissue and disease specificity, they projected the expression signatures onto occipital cortex (*n* = 45) and cerebellum ALS samples (*n* = 128), and motor cortex samples of controls (*n* = 59), showing that the subtypes were largely restricted to motor cortex and pwALS, with logistic regression-based classifiers achieving excellent discrimination between motor cortex, the other brain regions, and between cases and controls. Unlike earlier studies that incorporated both gene and TE expression counts into a combined matrix for clustering, this study derived subtypes solely from gene expression data, demonstrating that the molecular signatures are robust even without TE features. They also examined whether the subtypes could be detected in peripheral blood from pwALS, applying the model to RNA sequencing (RNA-seq) data from peripheral blood mononuclear cells (*n* = 15) and microarray data from whole blood (*n* = 397). While separation was less distinct than in brain tissue, they reported high assignment probabilities (80–90%) across all three groups, suggesting that the subtypes are at least partially recapitulated in blood and may provide a foundation as accessible biomarkers for patient stratification.

**Fig. 2:**
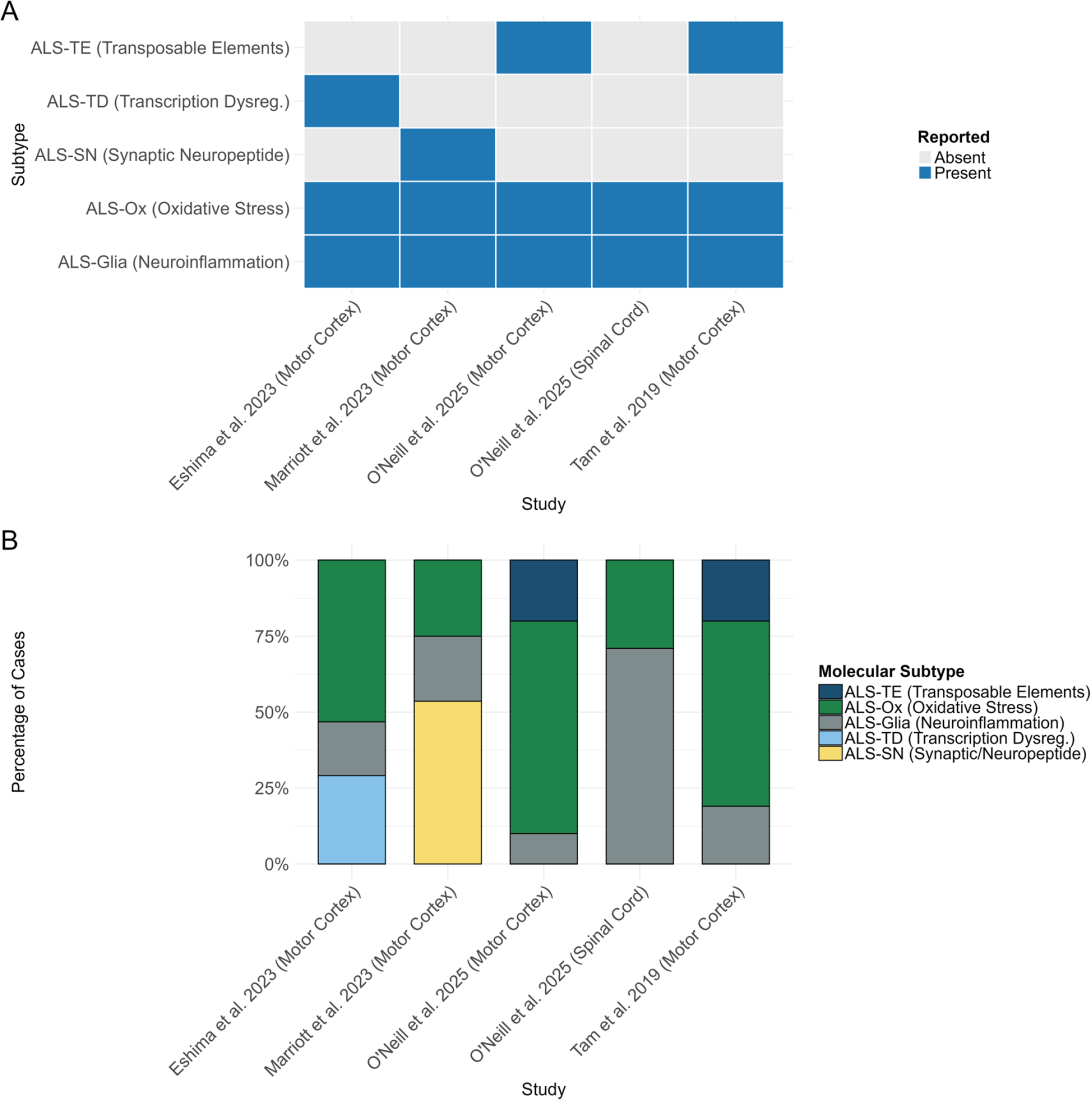
Reported ALS molecular subtypes in the nervous tissue across studies. **A** Heatmap showing whether each subtype was identified in Eshima et al. [10], Marriott et al. [11], O’Neill et al. [12], and Tam et al. [13]. **B** Relative prevalence of subtypes reported in each study. Across datasets, ALS-Glia and ALS-Ox subtypes are consistently observed, while ALS-TE and ALS-TD appear more study- and tissue-specific. ALS-SN was uniquely described by Marriott et al. [11]

In 2025 [12], a team of scientists from the same lab as that performing the initial 2019 study built upon their original findings by training a deep ALS neural net classifier (DANCer) on their NYGC dataset in order to generate a classifier of subtype assignment able to identify the ALS subtype in the newly released expanded cohort from the NYGC ALS Consortium. Furthermore, to determine whether the subtypes were present in this new cohort they re-ran a non-smooth non-negative matrix factorisation (nsNMF) model with the new independent samples and identified 3 clusters consistent with their original findings, i.e. ALS-Ox, ALS-Glia, and ALS-TE. Crucially, this study compared molecular subtypes between post-mortem motor cortex and spinal cord, analysing 664 ALS and control samples. Within this dataset, 267 individuals with ALS had both motor cortex and spinal cord available, allowing direct comparison of subtype distributions within the same donors. The analysis revealed noticeable tissue-specific differences, where ALS-Glia represented the smallest group in cortex (10%) but the majority in spinal cord (71%), while the ALS-TE subtype was absent from spinal cord (Fig. 2B). Moreover, disease duration was found to correlate with TDP-43 dysfunction in the motor cortex and with inflammatory signatures in spinal cord, highlighting the clinical relevance of these transcriptomic subtypes.

### Brain molecular subtypes and clinical relevance

#### Consistency of the molecular subtypes across studies

Across all studies exploring and identifying the molecular subtypes in ALS brain samples, a total of 5 have been identified across various cohorts (Fig. 2A). This includes ALS-Ox, ALS-Glia, ALS-TE, ALS-TD, and ALS synaptic and neuropeptide signalling (ALS-SN) [10–13]. Noticeably, both ALS-Ox and ALS-Glia were established in all studies and by all teams whereas ALS-TE, ALS-TD, and ALS-SN were only identified by individual teams (Fig. 2A). The 2023 study [10] identified the ALS-TD subtype, which displays molecular pathways broadly consistent with those originally observed in ALS-TE suggesting potential mechanistic overlap between transcriptional dysregulation and transposable element activation. However, while TE expression characterised only the ALS-TE subtype, the expression of individual TEs was a characteristic of both ALS-TD and ALS-Ox in the later study. The biological relevance of most identified subtypes is supported by molecular mechanisms that are well-established in the literature exploring ALS pathogenesis. Each subtype demonstrates distinct pathological processes, including oxidative stress (ALS-Ox) involving mitochondrial dysfunction and reactive oxygen species accumulation, consistent with decades of research demonstrating oxidative stress as a core pathogenic mechanism in ALS, particularly in SOD1-associated disease where mutant SOD1 was known to generate toxic reactive oxygen species [19, 20]; glial activation and neuroinflammation (ALS-Glia) characterised by microglial and astrocytic dysfunction, validating extensive prior evidence of neuroinflammation in ALS including early PET imaging studies showing microglial activation and neuropathological findings of reactive astrogliosis in post-mortem tissues [21, 22]; transcriptional disruption (ALS-TD) involving RNA-binding protein dysfunction, directly confirming the central role of RNA metabolism dysfunction established through discoveries of TDP-43, *FUS*, and *C9orf72* mutations, all of which encode RNA-binding proteins [23]; and altered neuropeptide signalling (ALS-SN) affecting motor neuron survival and synaptic function, aligning with emerging evidence of synaptic dysfunction and altered neurotrophic signalling in ALS, extending beyond the traditional focus on motor neuron cell death [24, 25]. Transposable element dysregulation (ALS-TE) represented a novel pathogenic mechanism not previously recognised in ALS, though it had been linked to other neurodegenerative diseases and aging [26, 27]. The 2019 study demonstrated that TEs are linked to TDP-43 pathology and genomic instability in vitro and in vivo.

##### ALS-TE: increase in transposable element expression subtype

The ALS-TE subtype was identified by Tam et al. [13] and subsequently reproduced by O’Neill et al. [12] with both studies leveraging the NYGC ALS consortium dataset and overlapping teams. The subtype represents approximately 20% of ALS across both respective studies (Table 1; Fig. 2B). Using single-cell analysis of frontal and motor cortex tissue, O’Neill et al. [12] were able to demonstrate greater evidence of TDP-43 dysfunction in motor neurons, elevated TE expression in astrocytes, and increased enrichment of microglia in homeostatic clusters for samples belonging to this phenotype. ALS-TE is also characterised by the increased expression of TE and TDP-43 dysfunction, with key molecular features including aberrant retrotransposon activation, dysregulated RNA processing, and increased pseudogene expression [12, 13]. Regarding the prognostic correlations of this subtype, O’Neill et al. [12] identified that individuals within this subgroup have a significant negative correlation with disease duration (*r* = − 0.25, *p* < 0.05), indicating shorter survival times, though the specific magnitude of this difference was not quantified. However, Tam et al. [13] were unable to establish this correlation but did identify an association with limb onset.

The identification of the ALS-TE subtype reinforced the previous notion of the potential association of retroviruses in ALS pathogenesis. Research dating back between the 1990s and early 2000s explored the possibility of retroviral involvement in ALS pathology due to elevated levels of viral antibodies and reverse transcriptase in serum belonging to pwALS, with many clinical trials exploring the use of antiretroviral therapies [28–31]. However, a study conducted in 2011 established the association of the Human endogenous retrovirus K (HERV-K) in pwALS, which is the first study to link an association of a TE species to ALS [32]. A later clinical trial study in 2019 explored the potential safety and tolerability of the long-term use of Triumeq, an antiretroviral drug capable of targeting retroviral reverse transcriptase [33]. They established that the long-term use of Triumeq was safe for pwALS and also found a decrease in ALSFRS-R scores by 21.8%. Although the results seemed promising, further molecular and mechanistic evidence was needed to establish a more holistic assumption that TE expression and mechanisms contribute to the disease. TE dysregulation had not been previously recognised as a major disease mechanism in ALS, despite being implicated in ageing and other neurodegenerative disorders [20, 34]. The discovery of the TE subtype in ALS provided the first systematic evidence that genomic instability through retrotransposon activation constitutes a distinct pathogenic pathway in a substantial subset of ALS patients [13]. The finding directly supported emerging hypotheses about TDP-43’s role in genomic instability, demonstrating that loss of TDP-43 nuclear function leads to a widespread increase in transposable element expression [20, 26].

##### ALS-Ox: oxidative stress subtype

The ALS-Ox subtype was concordant across all studies and displayed the largest proportion of pwALS, representing 25–70% of cases within their respective cohorts (Table 1). This subtype is characterised by an increase in oxidative stress, proteotoxic stress and mitochondrial dysfunction. Its molecular signatures include upregulation of *SOD1*, *OXR1* and *NEFL*, and altered oxidative phosphorylation [10–13]. Using single-cell analysis of frontal and motor cortex tissue, O’Neill et al. [12] identified an upregulation in genes associated with autophagy and lysosome activity in motor neurons. Similarly, Marriott et al. [11] identified a higher composition of astrocytes and endothelial cells within this subtype using cell deconvolution analysis, while Eshima et al. [10] found it associated with excitatory and inhibitory neurons using the same approach. Furthermore, patients belonging to this group displayed intermediate survival outcomes (36 months) and an age of onset (60 ± 1.16 years), as shown in Table 1 [10].

##### ALS-Glia: neuroinflammation/glial activation subtype

Individuals belonging to the ALS-Glia subtype represented the smallest subgroup across all studies, ranging from 10% to 21.4% [10–13]. This specific subtype is characterised by increased activity in neuroinflammatory signatures and glial cell activation. Cell composition analyses consistently revealed increased proportions of microglia, oligodendrocytes, glial progenitors, and vascular cells in samples associated with this subtype [10, 11, 13]. Additional evidence from single-cell data further supports microglial enrichment and activation [12]. This subtype correlates with the poorest prognosis (28 months median survival), and the latest age of onset (63.2 ± 1.83 years), suggesting an accentuated neuroinflammation may associate with an aggressive disease progression (Table 1) [10, 12]. The identification of the ALS-Glia subtype supported extensive prior evidence of neuroinflammation in ALS, including early PET imaging studies demonstrating microglial activation and neuropathological findings of reactive astrogliosis in post-mortem tissues [21]. This subtype has significantly impacted the field by reinforcing the central role of neuroinflammation in ALS pathogenesis and highlighting potential therapeutic targets focused on modulating microglial activation and inflammatory cascades, establishing neuroinflammation as a key driver of disease progression alongside traditional motor neuron degeneration paradigms [22, 35–37].

##### ALS-TD: transcription dysregulation in ALS

The ALS-TD subgroup was only identified in the study conducted by Eshima et al. [10]; representing 29.1% of the study’s sample cohort. Individuals in this subtype are more prone to transcription dysregulation, which is characterised by the aberrant expression of pseudogenes, intronic regions, and long non-coding RNA (Table 1). However, although this subtype shares similar pathways with the subtype ALS-TE, samples in this cluster did not exhibit TDP-43 dysfunction, suggesting dysregulation of alternative underlying mechanisms that govern transcription processes. The study was unable to identify any significant differences between the ALS-TD phenotype and cell composition but was able to establish that individuals with this phenotype have a more favourable prognosis (median survival: 42 months, Table 1), suggesting the underlying pathogenic mechanisms may exhibit less aggressive features than other subtypes [10].

##### ALS-SN: synaptic and neuropeptide signalling subtype

The ALS-SN subgroup was only identified in the study conducted by Marriott et al. [11]. It represented the highest proportion of samples within the study at a rate of 53.6% (Table 1). This subtype is characterised by the enrichment of pathways associated with neuronal and synaptic signalling, specifically related to processes involved in binding and receptor interaction, cAMP and neuroactive ligand transcription pathways, and neuropeptide activity. Through deconvolution analysis, motor neurons appeared to be more enriched in this subtype when compared to the other phenotypes [11]. However, the study was unable to establish a correlation between this phenotype and age of onset or disease duration, although it did report an association with inflammatory processes. Importantly, this analysis did not incorporate TE data, and therefore the presence of subtypes such as ALS-TE could not be assessed. Future studies that integrate TE expression data may help to confirm the existence of additional subtypes beyond ALS-SN.

#### Tissue specific differences in molecular signatures

The study conducted by O’Neill et al. [12] also explored the composition of subgroups between the frontal and motor cortex tissue with spinal cord tissue. They identified significant regional differences existing in subtype distribution and features. In spinal cord tissue, ALS-Glia predominates (71% vs. 29% for ALS-Ox), contrasting with motor cortex where ALS-Ox is more common (Fig. 2A). Spinal cord ALS-Glia samples show stronger TDP-43 splicing defects, while cortical ALS-TE samples exhibit more pronounced TE expression (Table 1). Importantly, these tissue-specific differences can occur even within the same individuals, with analysis of 267 patients having both motor cortex and spinal cord samples showing that the majority display different molecular subtypes between tissues [12]. This finding suggests that ALS molecular subtypes are not fixed for each patient but can vary across different tissues, highlighting the regional heterogeneity of ALS pathology and the need to consider tissue context when classifying subtypes.

### Biomarkers of molecular subtype

Although transcriptomic subtypes were initially defined in post-mortem motor cortex tissue, two studies have investigated whether they can also be identified pre-mortem in peripheral samples and thus serve as biomarkers. Grima et al. [14] investigated both diagnostic and prognostic transcriptomic biomarkers, as well as molecular subtypes, in peripheral blood from individuals with sporadic ALS (sALS). For diagnostic biomarkers, the authors identified 245 genes differentially expressed between sALS cases and controls and also identified 6 genes exhibiting differentially expressed isoforms. These features were used to train a Random Forest Classification model which was able to discriminate sALS from controls with 78% accuracy. The study also explored the potential of prognostic biomarkers in peripheral blood samples. They performed weighted correlation network analysis to determine if co-expressed genes correlated with clinical features and were able to identify an association between 3 co-expressed gene modules with age at sample collection and age at disease onset. They also identified a single module (consisting of 25 genes) associated with sex differences; these enriched genes are characterised to be involved in bacterial defence response. However, the study was unable to establish a correlation between predicted disease duration and actual disease duration between participants using the gene expression profiles. The study also explored two forms of patient stratification to investigate the molecular heterogeneity between subgroups and to identify relevant biomarkers. They first analysed the differences in gene expression between early and late-stage patients when compared to controls and were able to identify 279 differentially expressed genes between late-stage sALS cases and controls but were only able to identify a single differentially expressed gene (*C4BPA*) between early stage sALS and controls. Separately, they applied unsupervised clustering analysis, which revealed four distinct patient clusters with divergent gene-expression profiles, supporting the presence of underlying molecular heterogeneity among people with sALS. These findings imply that peripheral transcriptomic profiles could reflect disease subtypes, though further validation is required to determine whether such clustering corresponds to previously defined cortical subtypes or holds prognostic value across independent cohorts.

Marriott et al. [11] also trained subtype classifiers using motor cortex bulk RNA-seq with nsNMF and then projected these signatures onto peripheral blood datasets, including PBMC RNA-seq and whole-blood microarrays. They reported that ALS-Ox, ALS-Glia, and ALS-SN subtypes could be detected in blood with high assignment probabilities, although the degree of separation between groups was weaker than that observed in the cortex tissue. This study provided the first evidence that molecular programmes derived from post-mortem motor cortex tissue are at least partially preserved in blood, supporting their potential as minimally invasive biomarkers.

Catanese et al. [38], as part of a larger study whose primary aim was to investigate the transcriptional landscape of the main genetic subtype of ALS (*C9orf72* expansion), investigated the presence of ALS molecular expression signatures in blood and their relationship with those in the central nervous system (CNS) samples. They first used a deep learning model to identify an expression signature in a large, Dutch dataset of expression data from blood samples (397 ALS patients and 645 controls) [16] that was able to discriminate between case and control samples. The resulting gene set was enriched for genes related to the toll-like receptor (TLR) cascade, immune response, and autophagy. By overlapping and analysing their blood signature with other expression datasets of CNS samples and hiPSC-derived motor neurons from pwALS carrying *C9orf72*, *FUS*, *SOD1*, and *TARDBP* mutations, they showed common transcriptional alterations across all tissues and samples underlying and discriminating ALS from control samples. Although this study focussed on the main genetic sub-type of ALS and the transcriptional differences between ALS and controls, it provided strong evidence of the possibility to identify ML derived signatures of ALS that are consistent, at least partially, across ante-mortem and post-mortem samples.

Eshima et al. [10] added further complexity to the cross-tissue exploration of the subtype transcriptional signatures by investigating the consistency of the molecular subtypes across motor cortex and spinal cord samples of the same individuals. They found that subtype assignments could vary depending on the region analysed from the same individual, with ALS-Glia more prominent in spinal cord and ALS-TE more detectable in motor cortex. Although this study did not extend directly to blood, the results emphasise that although the expression of some of the genes that make up an individual subtype signature might be preserved across different tissues, the overall subtype identification is strongly tissue-dependent, raising important challenges for developing consistent peripheral biomarkers.

More recently, O’Neill et al. [12] applied single-nucleus RNA-seq across the motor cortex, spinal cord, and cerebellum and explored whether molecular subtypes defined in CNS tissue could be reflected in peripheral blood. They found that signatures associated with ALS-Ox and ALS-Glia could indeed be identified in blood transcriptomes, supporting the idea that these subtypes manifest systemically. However, they also reported that transcriptional programmes were often region-specific, and subtype assignment could vary depending on the tissue examined from the same donor. This observation reinforces the tissue dependence highlighted by Eshima et al. [10] and underscores both the promise and the challenge of developing reliable blood-based biomarkers.

Across these studies, certain signatures have shown stronger potential for ante-mortem detection. The oxidative phosphorylation and mitochondrial stress pathways characteristic of ALS-Ox were reproducibly observed in both cortical and peripheral datasets, suggesting systemic mitochondrial dysfunction. Similarly, major histocompatibility complex class II, complement activation, and interferon-stimulated modules associated with ALS-Glia were consistently detected in blood-derived immune cells, consistent with the immune-driven nature of this subtype. In contrast, features of ALS-TE and ALS-TD, such as transposable element activation and cryptic exon inclusion, remain largely confined to cortical tissue, with no robust demonstration in blood to date. Signatures linked to ALS-SN, including synaptic signalling and axon guidance, were strongly represented in motor cortex but were less clearly observed in peripheral samples [11]. Taken together, these studies indicate that pre-mortem detection of molecular subtypes is feasible, particularly for ALS-Ox and ALS-Glia, and that blood-derived transcriptomic data can stratify patients into biologically meaningful groups. However, reproducibility and resolution of tissue outside of CNS remain limited, and some subtypes, such as ALS-TE and ALS-TD, have yet to be reliably identified in blood. Prospective validation in independent, longitudinal cohorts is therefore essential before molecular subtypes can be translated into clinically actionable biomarkers, and these findings highlight their potential utility for stratified clinical trial design and patient enrichment strategies [10–12, 38].

## Conclusions

In this review, we focus on studies that aimed to identify the molecular subtypes of ALS utilising unsupervised clustering and transcriptomic data. Notably, the reviewed studies have identified overlapping subtype classifications. The ALS-Ox and ALS-Glia subtypes were consistently identified across all studies reviewed, while the ALS-TE, ALS-TD, and ALS-SN subgroups were reported only by individual teams, most likely due to the technical and methodological differences implemented in their studies [10–13]. The characterisation and distribution of these subtypes amongst the ALS population highlight the heterogeneity of the disease and align with the underlying mechanisms that govern its pathology and development [15, 19–24, 26, 39]. However, it is important to note that while these studies appear to validate findings across different research groups, three studies utilised overlapping samples from the NYGC ALS Consortium dataset and two studies were from the same group [10, 12, 13]. This sample overall is largely due to the limited availability of suitable datasets in ALS and may be a limiting factor for independent validation due to population bias and other generalisability concerns. However, while there is some overlap in sample sources, each study did apply its own distinct clustering frameworks while also investigating their robustness against independent studies. For instance, Eshima et al. [10] validated their findings using an independent study provided by Prudencio et al. [40]. This cross-cohort consistency strengthens the evidence for reproducible and biologically meaningful ALS subtypes. Another notable observation is the different molecular subtype patterns identified between tissue samples from O’Neill et al. [12] study. These findings also highlight regional vulnerability to specific pathological mechanisms and suggest that anatomically targeted therapeutic approaches may be necessary [12].

Furthermore, the studies also explored the clinical relevance for each subtype and found correlations with survival duration, age of onset, and with some subtypes having an association with limb onset and inflammatory processes. These findings highlight their potential use as prognostic markers and clinical stratification. Although not yet implemented in routine clinical practice, their associations with key clinical features and molecular mechanisms suggest they could be used to stratify patients in clinical trials, guide personalised treatment approaches, or serve as targets for therapeutic intervention. To this end, some of the studies explored the possibility of identifying subtypes in transcriptomic data from blood samples of pwALS. They showed that subtype signatures derived from the CNS can be used to classify pwALS into these subtypes using blood-derived data (Marriott et al.) and that the expression of key gene markers is consistent between ALS blood and brain samples (Catanese et al.). One study, Grima et al. [14], focussed on deriving the subtypes directly from whole-blood RNA-seq or pwALS and identified four patient subgroups (called cluster 0–3 by the authors) with distinct molecular characteristics. While these de novo blood clusters were not explicitly aligned with the cortical subtypes, their biology partly overlaps consistently with the findings from the other studies. Immune/inflammatory signatures in cluster 0 display similar characteristics to that of glial activation or neuroinflammation that defines ALS-Glia in brain tissue, indicating the relevance of immune activity in ALS pathogenesis. In converse, the proteostasis and RNA processing differences that distinguish clusters 1 and 2 resemble similar overlap between oxidative or proteotoxic stress of ALS-Ox and the RNA dysregulation seen in ALS-TD, respectively.

However, due to the lack of matching blood and brain samples from the same individuals, none of the studies could establish reliable blood biomarkers or investigate whether matching brain and blood samples correspond to the same subtype in pwALS. Investigating this issue is particularly important given the results from O’Neill et al., which demonstrated that samples from the same individual, but from different motor tissues, might belong to different subtypes.

Some of the studies also explored whether expression signatures could be used to discriminate between samples obtained from pwALS and non-affected controls. Marriott et al. showed that the same genes comprising the signature of the ALS subtype can distinguish brain samples of pwALS from non-affected controls with high accuracy (AUC 0.88). Using RNA-seq data from blood samples of pwALS and controls, Grima et al. trained a machine learning classifier with high discriminatory power (accuracy 78%). Similarly, Catanese and colleagues developed a deep learning classifier using microarray data and demonstrated that the expression of key ALS genes underpinning the classifier is conserved across all ALS samples and correlates between blood and CNS samples. These results highlight the diagnostic potential of these approaches beyond their use in patient stratification.

In conclusion, the reviewed studies provide convincing evidence that molecular subtypes exist in ALS samples from both CNS and peripheral tissues, and that machine learning can identify their signatures in transcriptomic data. These subtypes and their expression footprints offer great potential for developing transformative approaches to patient clinical and biological stratification, as well as diagnostics. However, further investigation into their generalizability and usability using large, independent datasets, comprising multiple matching CNS and peripheral samples, as well as longitudinally collected biofluids from the same individuals, is crucial to establish a definitive re-classification of ALS subtypes, identify viable biomarkers, and understand their evolution throughout disease progression and across tissue types.

## Supplementary Information

Additional file 1: Supplementary Materials.

## Data Availability

No new datasets were generated or analysed in this study. All data discussed are derived from previously published studies, which are cited in the manuscript and summarised in the accompanying tables.

## Abbreviations

ALS: Amyotrophic lateral sclerosis
ML: Machine learning
TE: Transposable element
ALS-TE: ALS transposable-element subtype
ALS-Ox: ALS oxidative-stress subtype
ALS-Glia: ALS glial-activation subtype
ALS-TD: ALS transcription-dysregulation subtype
ALS-SN: ALS synaptic/neuropeptide-signalling subtype
TDP-43: TAR DNA-binding protein 43
pwALS: People with ALS
sALS: Sporadic ALS
PBMC: Peripheral blood mononuclear cell
RNA-seq: RNA sequencing
nsNMF: Non-smooth non-negative matrix factorisation
NMF: Non-negative matrix factorisation
TLR: Toll-like receptor
NMD: Nonsense-mediated decay
lncRNA: Long non-coding RNA
CNS: Central nervous system
