# Supplementary material for: Classification of ALS molecular subtypes: a literature review on machine learning applications and their clinical value
Source: BMC Med. 2026 Feb 23;24:183. doi: 10.1186/s12916-026-04725-y (PMC13037183; doi:10.1186/s12916-026-04725-y)
Supplement: Supplementary file 1 — Additional file 1: Supplementary Materials. [file 12916_2026_4725_MOESM1_ESM.docx]

**Additional Materials**

# 1 Biological Samples, Data Types and Processing

## **Biological Samples**

The type of biological sample required to explore molecular subtypes is critical in shaping the value of the proposed models. For example, whether data are generated from single-cell or bulk profiles, from pre- or post-mortem tissue, and from regions more or less affected by the disease will determine which molecular signals are captured, thereby shaping the subtypes that can be resolved and the apparent specificity of any biomarkers. In ALS, the motor cortex is one of the primary sites affected by disease pathogenesis, which ideally pins interest into identifying markers that could indicate disease onset or cause. Therefore, using the motor cortex in ALS research is the 'gold standard' when investigating pathological mechanisms, validating biomarkers, and confirming therapeutic targets (1, 2). As a result, the majority of the reviewed studies relied on data from post-mortem frontal and motor cortex tissues (Supp. Table 1) (3-6). While these samples offer valuable insights into end-stage disease processes, they also carry limitations, including sample degradation and the inability to track the progression of the disease in its earlier phases due to the invasiveness nature in collecting these samples (2). Nonetheless, some studies have explored other tissues; such as peripheral blood mononuclear cells (PBMCs) to potentially identify accessible biomarkers or perform *in vitro* studies using human induced pluripotent stem cells (hiPSCs) (Supp. Table 1) (1, 4, 7).

Blood-based biomarkers are widely applicable in both research and clinical settings, as they allow minimally invasive sampling and provide valuable longitudinal insights into disease progression. For example, Marriott et al., (4) demonstrated that RNA-seq data from PBMCs could readily discriminate individuals assigned to different molecular subtype groups. Similarly, Catanese et al. (7) showed that PBMCs from both sporadic and familial ALS patients capture peripheral signatures that reflect central disease processes, highlighting their promise as accessible biomarkers. Crucially, such approaches avoid the need for motor cortex tissue, making biomarker discovery more feasible given the ease of blood accessibility. hiPSCs also represent an innovative approach employed by Catanese, et al., (7), allowing for disease modelling in controlled laboratory conditions in vitro. These cellular models enable the study of early disease mechanisms that might not be apparent in post-mortem tissue.

## **Data Types and Processing**

All reviewed studies predominantly used short-read RNA-seq data to generate transcriptome profiles. This technology provides a comprehensive snapshot in time of gene expression patterns, capturing both protein-coding and non-coding RNA species. Identifying molecular subtypes using this type of data can be very advantageous as it can provide further insight into the metabolic mechanisms of the tissues investigated; allowing researchers to focus on the most variably expressed genes that provide meaningful metabolic patterns. Working with such data begins with the processing of raw RNA-seq data that would involve a series of data-refining stages including quality control, raw read alignment to a reference transcriptome, further data quality control, and generating count matrices containing an array of sample gene expression values used for downstream analysis (3-6). Other studies have also explored the use of whole genome sequencing (WGS); to explore the overall genome, and bisulfite sequencing (WGBS); to explore the epigenetic alterations that effect gene expression. Using this approach, Catanese, et al., (7) investigated the genomic and epigenomic alterations of ALS individuals. This further supports the value that applying multi-omic approaches can enable a more comprehensive understanding of the molecular subtypes of ALS. However, studies focusing on ALS molecular subtypes have typically applied unsupervised ML methods on these count matrices to cluster samples based on the variation of their gene expression values.

## **Identification of ALS Molecular Subtypes**

Across the reviewed studies, four applied unsupervised ML clustering methods to define molecular subtypes of ALS and examined their associations with age of onset and disease duration (3-6). These analyses drew on large-scale transcriptomic datasets from frontal cortex, motor cortex, and blood. Data sources included TargetALS, the New York Genome Center (NYGC) ALS Consortium, the King’s College London (KCL) Brain Bank, and a publicly available dataset from Prudencio, et al., (8). Notably, there was considerable overlap in the datasets used. For example, Eshima, et al., (3), O’Neill, et al., (5) and Tam, et al., (6) analysed data from the NYGC ALS Consortium.

# 2 Computational approaches for the analysis of the ALS Transcriptome

## **Unsupervised Machine Learning and Cluster Characterisation**

Several studies in this review have employed unsupervised ML methods that specifically use the clustering of gene expression data amongst the study cohort. Applying this method on transcriptome count matrices can classify ALS samples into various distinct groups amongst the overall cohort and therefore previewing a transcriptional landscape without any prior classification. The method most commonly used across studies investigating molecular subtypes was the use of NMF algorithms (Supp. Table 1; Fig. 1) (3-6). In summary, NMF is a dimensionality reduction technique that factorises a non-negative matrix (where all values are greater than or equal to 0) into two separate lower-rank non-negative matrices. For instance, given a matrix *A* with dimensions *m* ×*n*, NMF decomposes it into two smaller matrices *W* and *H*, such that *A ≈ WH*. Here, *W* (*m* × *k*) represents the basis matrix containing *k* reduced features or components, while *H* (*k* × *n*) contains the coefficients that describe how these features combine to reconstruct the original data (9). The application of NMF has been used across various disciplines and fields as it provides a parts-based, interpretable representation of data, often offering more meaningful insights than traditional PCA analysis (10). In the context of gene expression data, NMF reduces the dimensionality to a chosen *k* value, yielding an optimal set of features that characterise the resulting clusters. When the method is applied to transcriptomic expression data, they can identify both subgroups of samples with homogenous gene expression profiles and their expression signatures. Following sample clustering, studies using this approach either applied gene enrichment analysis (GEA) or gene set enrichment analysis (GSEA) to characterise the clusters identified and establish the pathways enriched in each cluster (11). GEA is an over-representation approach that tests whether predefined pathways or functional categories are disproportionately represented within a selected gene list, such as genes most informative for a cluster (12). For example, GEA might reveal that apoptosis-related genes are enriched among the top-ranking genes defining a cluster. In contrast, GSEA does not rely on a preselected list of genes. Instead, it ranks all genes by their association with the cluster or phenotype and evaluates whether genes from a given pathway are systematically biased toward the top (upregulated) or bottom (downregulated) of this ranking (11). For instance, GSEA might detect that oxidative phosphorylation genes are consistently upregulated across the ranked profile, implicating mitochondrial activity. This enrichment step is critical because it translates cluster-specific expression patterns into biologically meaningful subtypes. Studies using these approaches to define molecular subtypes have repeatedly converged on similar or identical subtypes across independent datasets, underscoring their robustness (Table 1) (3-6).

## **Supervised Machine Learning**

Although unsupervised ML clustering can reveal data-driven subtypes without prior assumptions, supervised ML approaches can complement this by testing whether those clusters are reproducible and predictive in independent datasets. By assigning cluster labels derived from unsupervised analysis, supervised classifiers can be trained to evaluate whether the same patterns can be accurately recovered. This not only validates the biological relevance of the clusters but also tests their predictive robustness and generalisability across populations, tissue types, and data modalities. A few studies have applied supervised classification models using subtype labels (or cases-control status) to train and evaluate their initial findings (Supp. Table 1; Fig.1). For example, Marriott et al., (4) applied Linear Discriminant Analysis (LDA) to both discriminate ALS cases from controls and to cross-validate identified molecular subtypes across independent cohorts. They reported high assignment probabilities of 80–90% when validating the three subtypes across the TargetALS motor cortex dataset (N = 93), Italian blood RNA-seq (N = 15), and Dutch blood microarray data (N = 397). To further test discriminative power, they built logistic regression models to separate ALS cases from controls using a subtype-specific gene signature, achieving a ROC AUC of 0.88 ± 0.10, with precision = 0.80 ± 0.13, recall = 0.82 ± 0.19, and F1-score = 0.79 ± 0.14. These findings highlight how supervised ML can be used to validate unsupervised clustering, supporting both the robustness of ALS molecular subtypes and their potential translational value as reproducible biomarkers.

## **Deep Learning Applications**

Unlike conventional machine learning approaches, which often rely on predefined features selected by researchers (for example, principal components or gene co-expression modules), deep learning methods automatically extract hierarchical features from the data itself. This makes them especially powerful when dealing with very large and complex datasets such as transcriptomes or multi-omic profiles. Deep learning is particularly appropriate when conventional ML models reach a ceiling in accuracy, or when the biological signal is expected to be highly nonlinear and subtle. In the context of ALS, these models are well suited for both classification tasks (such as assigning patients to molecular subtypes) and discovery tasks (such as identifying shared pathways across genetically diverse patients). Importantly, because deep neural networks can capture complex patterns across tissues and modalities, they hold promise for biomarker discovery, where reproducibility across independent datasets is essential.

Two studies effectively applied deep neural network classifiers as shown in Supp Figure 1 (5, 7). These models can be deployed in prediction mode to classify new samples not seen during training. For example, O’Neill et al., (5). For example, O’Neill, et al., (5)first used unsupervised non-negative matrix factorisation to identify and characterise three distinct molecular clusters within the ALS cohort (ALS-Ox, ALS-Glia, ALS-TE). They then developed the Deep ALS Neural net Classifier (DANCer), a supervised neural network, to validate these unsupervised ML findings and confirm the robustness of the clusters, achieving 96.4% classification accuracy on the labelled dataset. Crucially, DANCer also enabled efficient classification of additional RNA-seq samples in prediction mode without requiring retraining. A related deep-learning approach was applied by Catanese, et al., (7), who integrated blood and spinal cord transcriptomes using a multi-layer neural network via the Keras/TensorFlow R package; identifying convergent predictors enriched for toll-like receptor (TLR) signalling which was also consistent with signatures observed in hiPSC-derived motor neurons. They also extended their analysis to derive a deep-learning mutational signature for ALS, underscoring the potential of deep models to uncover common disease mechanisms despite underlying genetic heterogeneity.


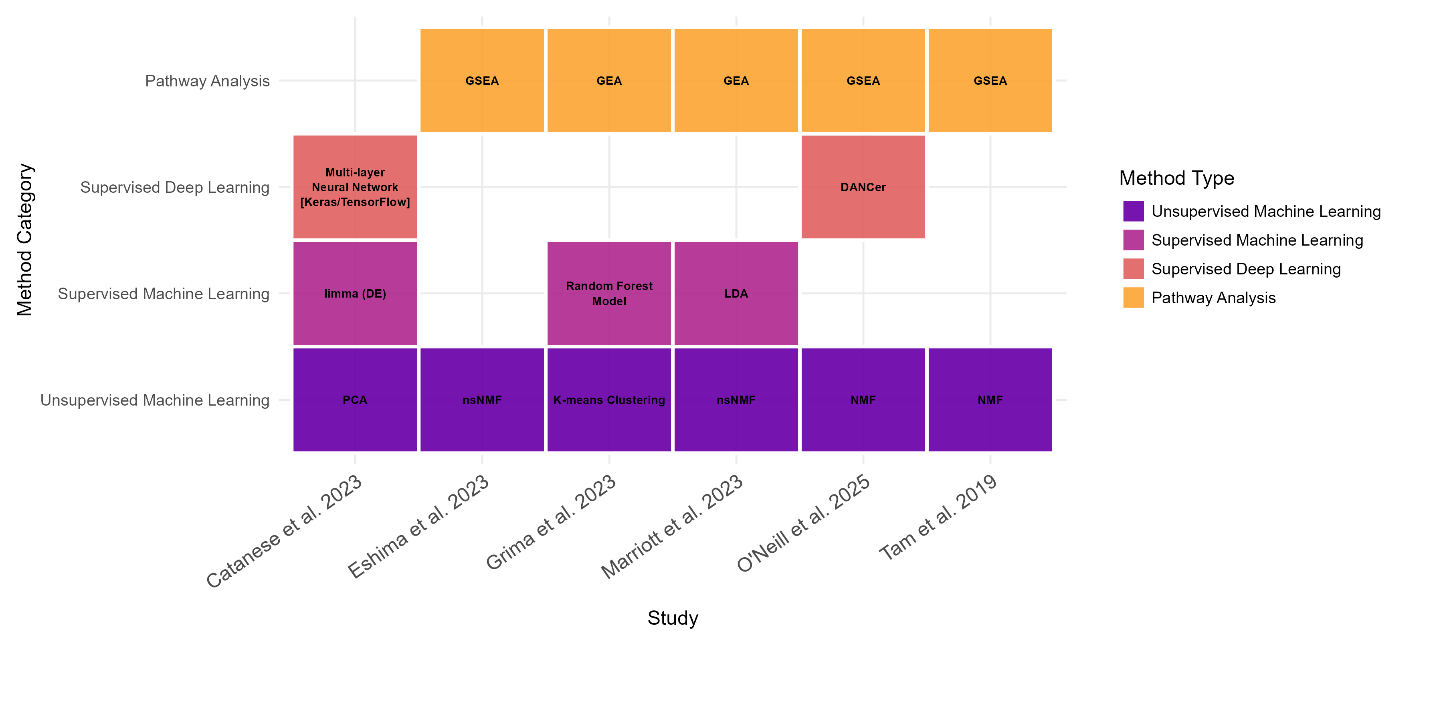


**Supplementary Figure 1.** Overview of the analytical approaches used in ALS transcriptomic subtype studies. Each block represents a methodological component categorised as unsupervised machine learning, supervised machine learning, supervised deep learning, or pathway analysis. Studies applied combinations of principal component analysis (PCA), K-means clustering, non-smooth non-negative matrix factorisation (nsNMF), and standard NMF, often alongside supervised methods (e.g. LDA, limma and a Random Forest model) or deep learning (e.g. DANCer). Pathway-based analyses, including gene set enrichment analysis (GSEA) and gene enrichment analysis (GEA), were frequently used to interpret subtype-associated biology. The diversity of approaches highlights the challenges in comparing results across studies.

| **Study**  **Supplementary Table 1: Analytical Methods and Data Types Used in Machine Learning-based Subtyping ALS Studies** | **Sample Type** | **Cohort Size** | **Analytical Method** | **Omics Type** |
| --- | --- | --- | --- | --- |
| Catanese, et al., 2023 | **Human Induced Pluripotent stem cells** | ALS Cases (n = 8)  Controls (n=3) | **RNA-seq analysis:**   - Differential expression analysis (R package limma) - PCA used to visualise a clear separation between case groups and healthy controls   **WGBS analysis:**   - Differential methylation analysis   Correlation between differential methylated regions (DMR) and differential expressed genes (DEG) | **Transcriptomics**  (Bulk RNA-seq)  **Methylomics**  **(WGBS)** |
|  | **Peripheral blood mononuclear cells** | sALS Cases (n=10)  fALS Cases (n=10)  Controls (n= 10) | - Differential expression analysis (R package limma) - Gene Set Enrichment Analysis | **Transcriptomics**  (Bulk RNA- seq) |
|  | **Whole Blood** | ALS Cases (n=397)  Controls (n=645) | Deep machine-learning (Keras/Tensorflow, multi-layer neural network) | **Transcriptomics**  (Bulk RNA- seq) |
|  | **Spinal cord** | ALS Cases (n=169)  Controls (n=57) | Deep machine-learning ( Keras/Tensorflow, multi-layer neural network ) | **Transcriptomics**  (Bulk RNA- seq) |
|  | **Whole genome** | ALS Cases (n=773)  Controls (n=93) | Generated 96 non-negative matrix factorised based somatic signatures | **Whole genome sequencing** |
| Eshima, et al., 2023 | **Post-mortem frontal and motor cortex tissue** | ALS Cases (n=208; tissue samples (n=451))  FTLD Cases (n=42)  Controls (n=93) | Unsupervised clustering using non-smooth non-negative matrix factorization (nsNMF); used in SAKE tool  Gene Set Enrichment Analysis | **Transcriptomics**  (Bulk RNA- seq) |
|  | **Clinical data (age at onset, survival, etc.)** | ALS Cases (n=208) | Kaplan-Meier survival analysis | Patient clinical parameters |
| Grima, et al., 2023 | **Whole Peripheral Blood** | sALS Cases (n=96)  Controls (n=48) | - Differential expression analysis (R package edgeR) - Differential transcript usage analysis (R package IsoformsSwitchAnalyzeR) - Principle component analysis for clustering - Supervised machine learning (Random Forest classifier model) | **Transcriptomics**  (Bulk RNA- seq) |
| Marriott, et al., 2023 | **Post-mortem motor cortex tissue** | **KCL Brain Bank:**  ALS Cases (n=112)  Controls (n=59) | Hierarchical clustering using non-smooth negative factorization (nsNMF) | **Transcriptomics**  (Bulk RNA- seq) |
|  | **Post-mortem Motor cortex (validation set)** | **Target ALS:**  sALS Cases (n=93; tissue samples (n=163)) | Linear discriminant analysis (LDA) | **Transcriptomics**  (Bulk RNA- seq) |
|  | **Peripheral Blood mononuclear cell datasets** | **Zucca Cohort:**  sALS Cases (n=15)  **Van Rheenan:**  ALS Cases (n=397) | Linear discriminant analysis (LDA) | **Transcriptomics**  (Bulk RNA- seq) |
| O’Neill, et al., 2025 | **Post-mortem frontal and motor cortex** | ALS Cases (n=431; tissue samples (n=733)) | Deep ALS neural net classifier (DANCer) in prediction mode. (Visualised using PCA) | **Transcriptomics**  (Bulk RNA- seq) |
|  | **Spinal Cord** | **Samples (n=664):**   - ALS Cases (n=331) - Controls (n=75) | non-negative matrix factorization (NMF) | **Transcriptomics**  (Bulk RNA- seq) |
|  | **Post-mortem motor cortex** | ALS Cases (n=26; tissue samples (n=48))  Controls (n=14; tissue samples (n=733)) | Single cell mode Deep ALS neural net classifier (scDANCer) in prediction mode. | **Transcriptomics**  (Single Cell RNA- seq) |
| Tam, et al., 2019 | **Post-mortem frontal and motor cortex tissue** | ALS Cases (n=77; tissue samples (n=148)) Controls (n=18; tissue samples (n=28)) | De novo clustering using non-negative matrix factorization (NMF) via the SAKE package  Gene Set Enrichment Analysis | **Transcriptomics**  (Bulk RNA- seq) |

# References

1. Grima N, Liu S, Southwood D, Henden L, Smith A, Lee A, et al. RNA sequencing of peripheral blood in amyotrophic lateral sclerosis reveals distinct molecular subtypes: considerations for biomarker discovery. Neuropathol Appl Neurobiol. 2023;49(6).
2. Thakral S, Purohit P, Mishra R, Gupta V, Setia P. Impact of RNA stability and degradation in different tissues on determination of postmortem interval: a systematic review. Forensic Sci Int. 2023;349.
3. Eshima J, O’Connor SA, Marschall E, Bowser R, Plaisier CL, Smith BS. Molecular subtypes of ALS are associated with differences in patient prognosis. Nat Commun. 2023;14(1).
4. Marriott H, Kabiljo R, Hunt GP, Khleifat AA, Jones A, Troakes C, et al. Unsupervised machine learning identifies distinct ALS molecular subtypes in postmortem motor cortex and blood expression data. Acta Neuropathol Commun. 2023;11(1).
5. O’Neill K, Shaw R, Bolger I, Tam OH, Phatnani H, Gale Hammell M. ALS molecular subtypes are a combination of cellular and pathological features learned by deep multiomics classifiers. Cell Rep. 2025;44(3):115402.
6. Tam OH, Rozhkov NV, Shaw R, Kim D, Hubbard I, Fennessey S, et al. Postmortem cortex samples identify distinct molecular subtypes of ALS: retrotransposon activation, oxidative stress, and activated glia. Cell Rep. 2019;29(5):1164–77.e5.
7. Catanese A, Rajkumar S, Sommer D, Masrori P, Hersmus N, Van Damme P, et al. Multiomics and machine learning identify novel transcriptional and mutational signatures in amyotrophic lateral sclerosis. Brain. 2023;146(9):3770–82.
8. Prudencio M, Humphrey J, Pickles S, Brown AL, Hill SE, Kachergus JM, et al. Truncated stathmin-2 is a marker of TDP-43 pathology in frontotemporal dementia. J Clin Invest. 2020;130(11):6080–92.
9. Belyadi H, Haghighat A. Machine learning workflows and types. In: *Machine Learning Guide for Oil and Gas Using Python*. Houston: Gulf Professional Publishing; 2021. p. 97–123.
10. Guo YT, Li QQ, Liang CS. The rise of nonnegative matrix factorization: algorithms and applications. Inf Syst. 2024;123.
11. Subramanian A, Tamayo P, Mootha VK, Mukherjee S, Ebert BL, Gillette MA, et al. Gene set enrichment analysis: a knowledge-based approach for interpreting genome-wide expression profiles. Proc Natl Acad Sci U S A. 2005;102(43):15545–50.
12. Mathur R, Rotroff D, Ma J, Shojaie A, Motsinger-Reif A. Gene set analysis methods: a systematic comparison. BioData Min. 2018;11(1).
